# Soil salinity assessment of a natural pasture using remote sensing techniques in central Anatolia, Turkey

**DOI:** 10.1371/journal.pone.0266915

**Published:** 2022-04-18

**Authors:** Orhan Mete Kılıc, Mesut Budak, Elif Gunal, Nurullah Acır, Rares Halbac-Cotoara-Zamfir, Saleh Alfarraj, Mohammad Javed Ansari

**Affiliations:** 1 Department of Geography, Faculty of Arts and Sciences, Tokat Gaziosmanpaşa University, Tokat, Turkey; 2 Department of Soil Science and Plant Nutrition, Faculty of Agriculture, Siirt University, Siirt, Turkey; 3 Department of Soil Science and Plant Nutrition, Faculty of Agriculture, Tokat Gaziosmanpaşa University, Tokat, Turkey; 4 Department of Soil Science and Plant Nutrition, Faculty of Agriculture, Kırşehir Ahi Evran University, Kırşehir, Turkey; 5 Department of Overland Communication Ways, Foundations and Cadastral Survey, Politehnica University of Timisoara, Timisoara, Romania; 6 Zoology Department, College of Science, King Saud University, Riyadh, Saudi Arabia; 7 Department of Botany, Hindu College Moradabad, Mahatma Jyotiba Phule Rohilkhand University Bareilly, Bareilly, Uttar Pradesh, India; Duy Tan University, VIET NAM

## Abstract

Soil salinity is a major land degradation process reducing biological productivity in arid and semi-arid regions. Therefore, its effective monitoring and management is inevitable. Recent developments in remote sensing technology have made it possible to accurately identify and effectively monitor soil salinity. Hence, this study determined salinity levels of surface soils in 2650 ha agricultural and natural pastureland located in an arid region of central Anatolia, Turkey. The relationship between electrical conductivity (EC) values of 145 soil samples and the dataset created using Landsat 5 TM satellite image was investigated. Remote sensing dataset for 23 variables, including visible, near infrared (NIR) and short-wave infrared (SWIR) spectral ranges, salinity, and vegetation indices were created. The highest correlation between EC values and remote sensing dataset was obtained in SWIR1 band (r = -0.43). Linear regression analysis was used to reveal the relationship between six bands and indices selected from the variables with the highest correlations. Coefficient of determination (R^2^ = 0.19) results indicated that models obtained using satellite image did not provide reliable results in determining soil salinity. Microtopography is the major factor affecting spatial distribution of soil salinity and caused heterogeneous distribution of salts on surface soils. Differences in salt content of soils caused heterogeneous distribution of halophytes and led to spectral complexity. The dark colored slickpots in small-scale depressions are common features of sodic soils, which are responsible for spectral complexity. In addition, low spatial resolution of Landsat 5 TM images is another reason decreasing the reliability of models in determining soil salinity.

## Introduction

Soil salinity is an important land degradation and desertification process seriously affecting productivity and sustainability of lands [1–4]. It is a dynamic phenomenon that develops under natural and anthropogenic processes, especially in arid and semi-arid regions due to low rainfall, excessive evaporation of shallow groundwater and high soluble salt content [5]. In addition, soluble salts accumulate on vicinity of rooting depth using irrigation water with high soluble salts and excessive fertilizer application [3]. The salinity affects chemical and physical properties of soils and cause a significant decrease in the crop productivity [6–9].

The global coverage area of salt-affected soils is around 1060.1 million ha [10]. Salinity was reported on 1518722 ha of the land in Turkey, and saline soils constitute 5.48% of the total cultivable land of the country [11]. Reclamation of salt-affected lands is necessary for the effective and sustainable use of soil resources [12]. Therefore, accurate, cost-effective, and detailed soil salinity information is needed for the reclamation and management of saline lands [13–15].

High spatial variability of soil salinity over short distances requires the collection of numerous soil samples for accurate determination of salinity. Therefore, determining and monitoring soil salinity with classical methods (laboratory analysis) is costly and time-consuming. Unlike conventional methods, remote sensing technologies offer significant cost-effective advantages in determining soil salinity for large areas at high accuracy [5, 14, 16].

Remote sensing uses electromagnetic energy reflected from the earth surface to obtain information about earth. Recording the energy reflected from earth surface in different spectral ranges by sensors placed on satellites allows the detection and monitoring of differences on earth surface [17]. Several studies have been carried out to investigate and map saline areas by remote sensing through examining spectral characteristics of saline soils in visible (VIS) and infrared (IR) wavelengths [1, 14, 18, 19]. The basic approach in most of the studies was to develop regression models between ground sampling points and remote sensing datasets. Three different approaches, i.e., i) model development between reflection values of spectral bands in satellite images and measured soil salinity values [1, 20], ii) estimating soil salinity directly by calculating salinity indices [14, 19, 21] and iii) estimating soil salinity indirectly by calculating vegetation indices [14, 22, 23] are used to map soil salinity.

Soil salinity is detected and monitored in multiple scales using remote sensing methods due to the distinctive reflection of electromagnetic energy from salt crusts on soil surface [5, 19]. However, some factors such as soil texture, moisture, organic matter and iron oxide contents [24], and type and development period of vegetation [25] interfere the energy reflected or adsorbed from saline soils under natural conditions, which reduce the success in determining surface salinity by remote sensing. In addition, the resolution of the remote sensing images significantly affects the success in determining soil salinity by remote sensing [26–28]. Nevertheless, numerous studies have been published reporting that determining soil salinity using remote sensing has several advantages [4, 5, 19]. This perspective is accepted as a low-cost monitoring method of soil salinity, especially in basins with inadequate drainage [29, 30]. However, advances in remote sensing technology have not been able to offer an ideal data type and interrogation method combination to perform the same function for several environmental conditions in determining soil salinity [31, 32].

Salinity status of a land may change in a short period of time with the implementation of reclamation processes or changing the land use type [15]. Therefore, timely mapping of soil salinity through remote sensing is practical to monitor the changes in salinity level. Remote sensing have been used for a long time in soil salinity mapping in arid and semi-arid regions of the world. However, remote sensing data have been employed only in a few places of Turkey to map salinity [12, 33]. The current study is among the first studies determining soil salinity in agricultural lands and pastures using plant and soil indices by remote sensing. The main aim of the study was to investigate the potential of multispectral Landsat images for mapping surface soil salinity of a natural pastureland in an arid region of Turkey.

## Materials and methods

### Study area

**Fig 1:**
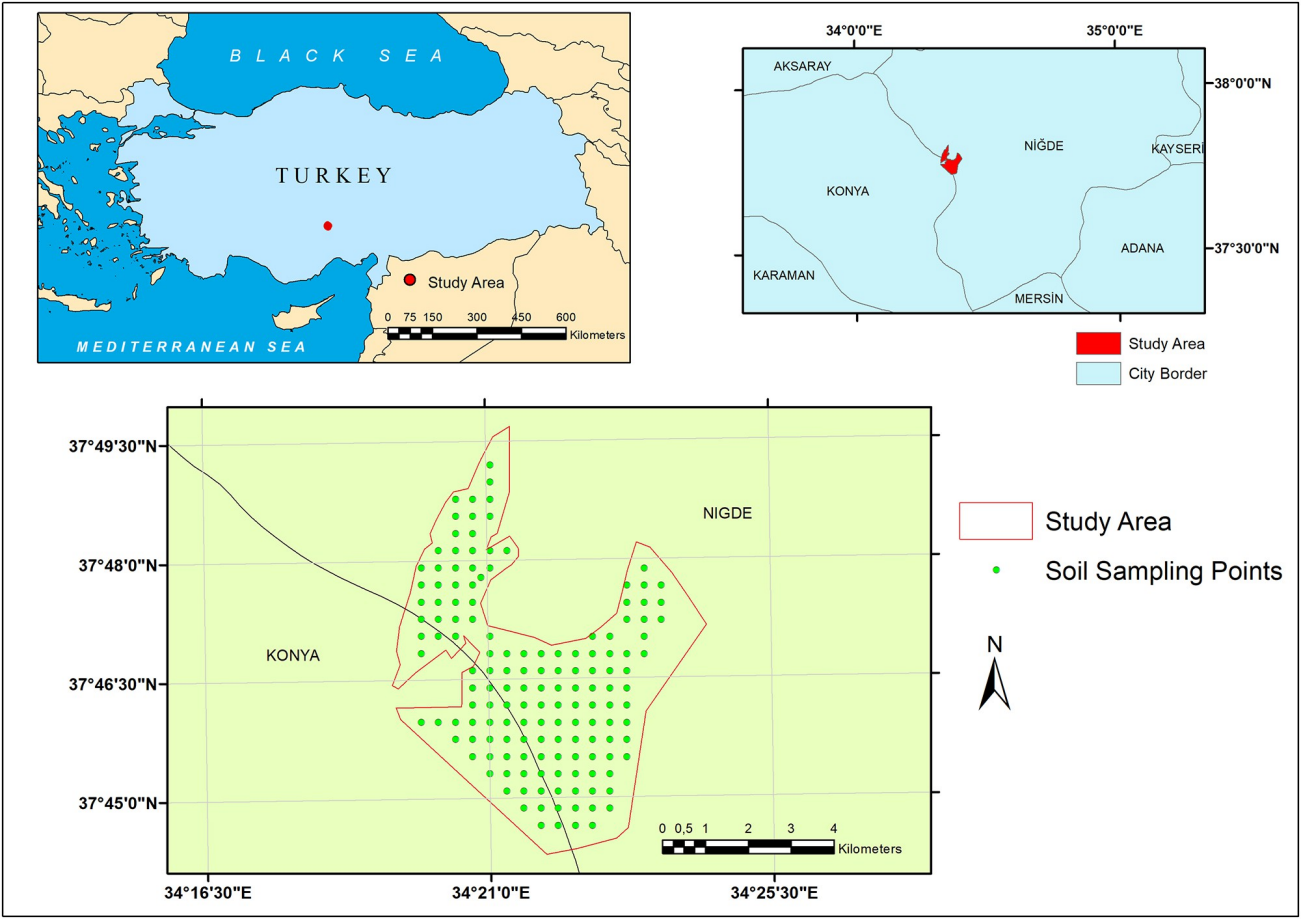
Location of the study area and soil sampling points.

The study area is situated in Emen Plain located in Nigde province, central Anatolia, Turkey between longitudes 34°16’30’’ and 34°25’30’’E and latitudes 37°49’30’’ and 37°45’30′′N (Fig 1). Total coverage area is 2650 ha pastureland. The area is characterized by arid conditions and long term (1935–2018) mean annual rainfall in Nigde province is 341.4 mm, while temperature is 11.2 °C. A new meteorological station was established within the study area during 2009 where elevation ranges from 1044 m to 1058 m, which is lower than the elevation of the long-term meteorological station (asl 1229 m). Mean annual rainfall between 2009 and 2019 was 220.6 mm.

**Fig 2:**
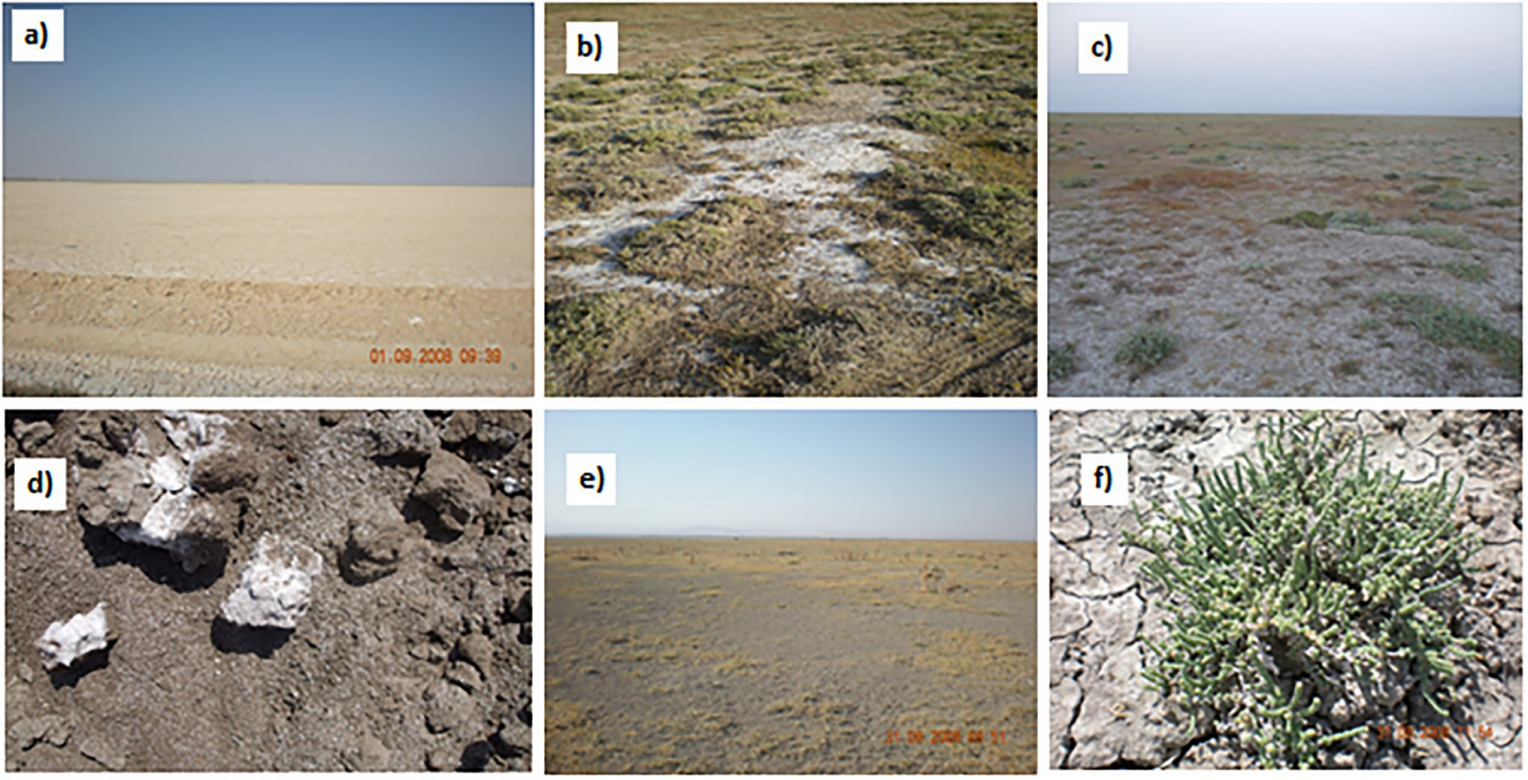
Saline areas with bare surfaces (a and b), area covered with salt crusts and halophytes (c), salt crystals on the surface and soil profile (d), surface with dried vegetation (e) and a sample halophyte plant (f). (Pictures taken by Mesut Budak).

Soil salinity in the study area varies from slightly to highly saline and NaCl is the dominant salt mineral [34]. The soil temperature regime calculated from the climate data recorded in the study area is mesic, and soil moisture regime is aridic [35]. The surface of the study area is covered with quaternary alluvium. Seven different soil series classified in Aridisol order have been defined in the detailed soil survey study [34]. The slope in study area is very low, which restricts drainage of surface water coming from surrounding mountainous areas. Surface water can only be removed from soil profile through evaporation, and this cycle has been repeated many times in the history. Each time salts brought by surface water accumulated on soil surface and created a severe salinity problem in the study area [13]. A significant portion of the land with high salt and sodium content has been classified as saline and sodic soil by Budak [34]. The study area contains mostly halophyte plants due to high salt content (Fig 2).

### Methodology

**Fig 3:**
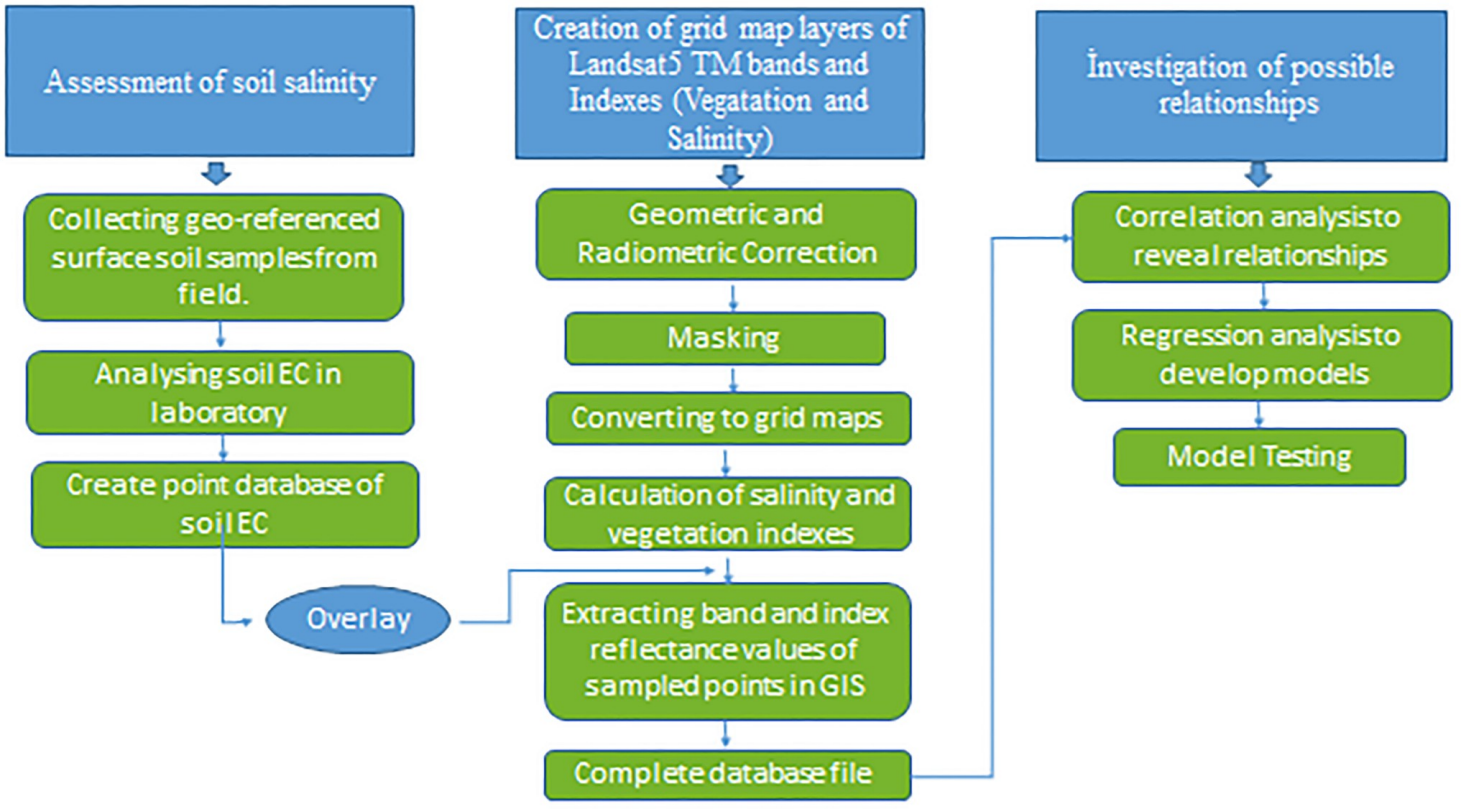
Flowchart of the applied methodology.

The methodology included three main steps, i.e., i) collection of soil samples from the study area and laboratory analysis to determine the electrical conductivity (EC), ii) determining reflectance values of Landsat 5 TM bands and calculation of the vegetation and salinity indices, and iii) determining the relations between the EC values of soil samples and the remote sensing dataset (Fig 3).

### Soil sampling and laboratory analysis

The study area (2650 ha) was divided into 400×400 m grids to collect soil samples. Total 145 soil samples were collected from 0–30 cm soil depth during June 2008, and the coordinates of the sample points were recorded using global positioning system (GPS) (Fig 1). Soil samples were dried at room temperature and then passed through a 2 mm sieve before laboratory analysis. The EC values of soil samples were measured in saturation pastes [36].

### Preparation of remote sensing database and statistical analysis

Soil samples were collected in 2008; therefore, the most appropriate satellite image at or close to this time was preferred. Landsat 7 ETM + satellite images started to suffer scan-line corrector failure as of 2003; thus, Landsat 5 TM image, which is the closest time to the date of soil sampling was preferred. Satellite images with the closest to the soil sampling date and the lowest cloud rate (Image Date acquired: 20.07.2008, path/row: 176/34) were downloaded from https://glovis.usgs.gov/ in Geotiff format. General characteristics of the downloaded image are given in Table 1.

**Table 1:** Characteristics of Landsat 5 TM bands.

Sensor	Band number	Band name	Wavelength (μm)	Resolution (m)
TM	1	Blue	0.45–0.52	30
TM	2	Green	0.52–0.60	30
TM	3	Red	0.63–0.69	30
TM	4	NIR	0.76–0.90	30
TM	5	SWIR 1	1.55–1.75	30
TM	6	Thermal	10.40–12.50	120
TM	7	SWIR 2	2.08–2.35	30

Digital numbers of the image were converted to reflection values by atmospheric and radiometric corrections to minimize atmospheric distortions in the satellite image and determine the real surface reflections [37]. Satellite image was divided into subsets by using the study area shapefile and the satellite image of the area was prepared for analysis. Twenty-three indices, including five Landsat 5 TM bands used in modeling process to determine soil salinity by remote sensing methods are given in Table 2. ERDAS-Imagine^®^ (version 2014) and SAGA GIS software were used to calculate the pre-processes and indices applied to the images [38].

**Table 2:** Equations used to analyze soil salinity and vegetation indices.

Salinity indices	Band ratios	Reference
Normalized difference salinity index	$NDSI=\frac{\;(R-NIR)}{\left(R+NIR\right)}$	[39]
Vegetation soil salinity index	VSSI = 2 x G– 5 x (R +NIR)	[40]
Brightness index	$BI=\sqrt{R^{2}+{NIR}^{2}}$	[39]
Salinity index-1	$SI=\sqrt{\left(BxR\right)}$	[39]
Salinity index-2	$SI=\sqrt{\left(GxR\right)}$	[39]
Salinity index-3	$SI=\sqrt{{(G}^{2}+R^{2}+{NIR}^{2}})$	[41]
Salinity index-4	$SI=\sqrt{{(G}^{2}+R^{2}})$	[41]
Salinity index-5	$SI=\frac{B}{R}$	[42]
Salinity index-6	$SI=\frac{\;(B-R)}{\left(B+R\right)}$	[42]
Salinity index-7	$SI=\frac{\;(GxR)}{B}$	[42]
Salinity index-8	$SI=\frac{\;(BxR)}{G}$	[43]
Salinity index-9	$SI=\frac{\;(NIRxR)}{G}$	[43]
Modified soil adjusted vegetation Index 2	${MSAVI}_{2}=(2NIR+1-\sqrt{{\left(2NIR+1\right)}^{2}-8(NIR-R))}/2$	[44]
Moisture stress index	$MSI=\frac{\;({SWIR}_{1})}{NIR}$	[45]
Normalized difference vegetation index	$NDVI=\frac{\;(NIR-R)}{\left(NIR+R\right)}$	[39]
Normalized difference water index	$NDWI=\frac{\;(NIR-SWIR1)}{\left(NIR+SWIR1\right)}$	[46]
Soil adjusted vegetation index (L = 0.5)	SAVI = (1 + L) × NIR −R/L + NIR + R	[40]
Transformed normalized difference vegetation index	$TNDVI=\sqrt{\frac{NIR-R}{\left(NIR+R\right)}+0.5)}$	[47]

Here, B = Blue, G = Green, R = Red, NIR = Near infrared, SWIR = Shortwave infrared

The bands of satellite image and the calculated indices were processed in GIS environment (ArcGIS 10.5) in grid format and overlaid with the prepared point database. A remote sensing dataset was created by extracting the reflection values of the Landsat TM bands and calculated indices using point database overlaid on the grid map layers. Linear regression analysis was used to investigate the relationship between measured EC values of soil samples and reflection values of satellite image and the calculated indices. Soil EC values were defined as dependent, and band and index reflection values were defined as independent variables. The distribution of data was tested out using the Kolmogorov-Smirnov test, which revealed that the EC values didn’t have a normal distribution. The results of normality for EC values are given in Table 3. The distribution of a variable is not considered normal if the significance value is lower than 0.05 in the Kolmogorov-Smirnov test (Table 3). The distribution of EC values was normalized using logarithmic transformation. The correlation and regression tests were applied following the logarithmic transformation process.

**Table 3:** Normality (Kolmogorov-Smirnov) test result of the soil EC and transformed soil EC.

Variable	Kolmogorov-Smirnov Test						
	Statistic	df	Sig.	Transformed variable	Statistic	df	Sig.
**EC**	0.148	145	0.000	LogEC	0.064	145	**0.200**

Pearson’s correlation test was used to determine the relationships between soil EC value and reflection values. The relationships between EC measurements of soil samples and developed indices and band reflection values were not strong enough. However, linear regression test was used to obtain models for some of the variables which had the highest correlations with the indices. The sensitivity of relationships was tested using variance analysis. The success of the prediction model in the variance analysis was assessed with the coefficient of determination (R^2^) value calculated by Eq 1. The variance analysis indicated that the sensitivity of relationships was not successful; thus, maps for the models were not produced. All statistical analysis were carried out using SPSS (version 22) software [48].

$$R^{2}=1-\frac{{\;\sum_{i=1}^{m}\left({\hat{y}}_{i}-y_{i}\right)}^{2}\;}{{\;\sum_{i=1}^{m}\left(y_{i}-\overset{-}{y}\right)}^{2}\;}$$ (1)

Where ${\hat{y}}_{i}$ is the observed value, $\overset{-}{y}$ is the predicted value and *y*_*i*_ is the mean of predicted value.

The model summary reveals the information whether correlation coefficient (r) between the observed and predicted values of dependent variable and determination coefficient (R^2^) in dependent variable can be explained with regression model. Large r and R^2^ values (close to 1) indicate a significant relationship and a reliable model, respectively [48]. Low values of r and R^2^ (close to 0) obtained in the study indicated poor relationships and a model that cannot be explained well, respectively. The minimal dataset of the study has been uploaded as S1 Dataset.

## Results

Descriptive statistics for soil properties in the study area are given in Table 4. Soils in the study area are mostly fine textured and mean clay, sand and silt contents were 52.40, 26.08 and 21.52%, respectively. The soils are mostly low in organic matter content, calcareous, saline, and sodic. Soil pH was between 7.51 and 9.31, electrical conductivity (EC) ranged between 0.61 and 27.40 dS m^-1^, exchangeable sodium percentage (ESP) was between 0.49 and 54.82% and sodium adsorption ratio (SAR) differed between 0.23 and 98.23. Exchangeable Na ranged from 0.55 to 76.18 me 100 g^-1^, with an average of 14.53 meq 100 g^-1^. Excess Na (ESP >15%; sodic soils) in surface soils caused dispersion of aggregates, closing-off soil pores and restricting water and air movement. Aggregate stability values within the study area varied between 7.91 and 99.72%, with an average of 73.72%. Slow water infiltration rate due to low aggregate stability resulted in waterlogging or perched water tables in these lands during and after rainy seasons [34]. In addition, sodic soils are known black alkali soils due to the dispersion and dissolution of humic substances, resulting in a dark color [49]. The color of soils with high ESP were darker despite their low organic matter contents [34] (Fig 2). The lime content varies between 3.99 and 49.47%, with an average of 31.43%, and organic matter content is between 0.32 and 4.50% with an average of 1.87%. The coefficient of variation values indicated that the variability of pH within the study area was low, the variability of lime, clay, silt, and aggregate stability were moderate, and SAR, exchangeable Na, EC, ESP, sand, and organic matter content were high.

**Table 4:** Descriptive statistics of surface soils (0–30 cm) in the study area [34].

	Min	Max	Mean	Std. Dev.	CV	Skewness	Kurtosis
Clay (%)	22.00	81.10	52.40	16.29	31.09	-0.124	-1.267
Sand (%)	3.87	61.55	26.08	14.24	54.58	0.432	-0.830
Silt (%)	8.75	55.11	21.52	6.45	29.97	0.672	2.557
Aggregate stability (%)	7.91	99.72	73.72	18.42	24.99	-0.787	0.412
pH	7.51	9.31	8.33	0.30	3.58	0.419	0.538
EC (dS/m)	0.61	27.40	6.09	5.50	90.45	1.328	1.502
CaCO_3_ (%)	3.99	49.47	31.43	10.81	34.40	-0.697	-0.604
Organic matter (%)	0.32	4.50	1.87	0.70	37.19	0.516	0.602
ESP (%)	0.49	54.82	12.71	11.31	88.98	1.277	1.220
SAR	0.23	98.23	14.07	14.80	105.19	2.138	5.938
Exc. Na (meq/100g)	0.55	76.18	14.53	14.38	98.99	1.403	1.689

Pearson correlation between LogEC, Landsat TM satellite bands and indices are given in Table 5. The correlations between 23 different indices and band reflection values, and LogEC values were not statistically significant. The LogEC values had positive correlations with independent NDWI, VSSI, SI5, SI6 and NDSI variables (0.421, 0.287, 0.231, 0.228 and 0.108, respectively), while negative correlations were recorded with other variables (Table 5). The highest negative correlation with LogEC values was found for SWIR1 band (-0.437), followed by MSI (-0.423), SI9 (-0.309) and NIR (-0.292) independent variables.

**Table 5:** Pearson correlation between soil EC and Landsat TM band, and index reflection values.

Variable	LogEC	Variable	LogEC
Blue	-0.188*	SI5	0.231**
Green	-0.202*	SI6	0.228**
Red	-0.247**	SI7	-0.233**
NIR	-0.292**	SI8	-0.251**
SWIR1	-0.437**	SI9	-0.309**
NDSI	0.108	MSAVI2	-0.167*
VSSI	0.287**	MSI	-0.423**
BI	-0.279**	NDVI	-0.108
SI1	-0.228**	NDWI	0.421**
SI2	-0.227**	SAVI	-0.154
SI3	-0.264**	TNDVI	-0.112
SI4	-0.230**		

** Correlation is significant at the 0.01 level,

* Correlation is significant at the 0.05 level.

Linear regression test was applied to obtain a model between soil salinity and Landsat 5 TM satellite bands (SWIR1 and NIR) and indices calculated from the image (MSI, NDWI, SI9 and VSSI), which had the highest correlation coefficients. The results for linear regression test performed between indices and band variables, and logEC values are presented as model summary (Table 6) and analysis of variance (Table 7).

**Table 6:** Summary of linear regressions for different models.

Model	r	R^2^	Adjusted R^2^	Standard Error of the Estimates
EC and SWIR	-0.437	0.191	0.186	0.369
EC and MSI	-0.423	0.179	0.173	0.371
EC and NDWI	0.421	0.178	0.172	0.372
EC and SI9	-0.309	0.095	0.089	0.390
EC and NIR	-0.292	0.085	0.079	0.392
EC and VSSI	0.287	0.082	0.076	0.393

**Table 7:** The results analysis of variance for linear regressions.

ANOVA		Sum of Squares	df	Mean square	F	Significance
SWIR and EC	Regression	4.605	1	4.605	33.802	0
	Residual	19.483	143	0.136		
	Total	24.088	144			
MSI and EC	Regression	4.303	1	4.303	31.100	0.000
	Residual	19.785	143	0.138		
	Total	24.088	144			
NDWI and EC	Regression	4.277	1	4.277	30.870	0.000
	Residual	19.812	143	0.139		
	Total	24.088	144			
SI9 and EC	Regression	2.299	1	2.299	15.084	0.000
	Residual	21.790	143	0.152		
	Total	24.088	144			
NIR and EC	Regression	2.050	1	2.050	13.301	0.000
	Residual	22.038	143	0.154		
	Total	24.088	144			
VSSI and EC	Regression	1.984	1	1.984	12.836	0.000
	Residual	22.104	143	0.155		
	Total	24.088	144			

Information on the regression outputs and the variation explained by the models are given in Table 7. The regression sum of squares values of the strong models should be larger than the residual sum of squares values. The results of ANOVA indicated that the indices don’t explain the majority of total soil salinity variation in the study area. Salinity and vegetation indices could not provide a strong model to distinguish soil salinity in the region.

## Discussion

**Fig 4:**
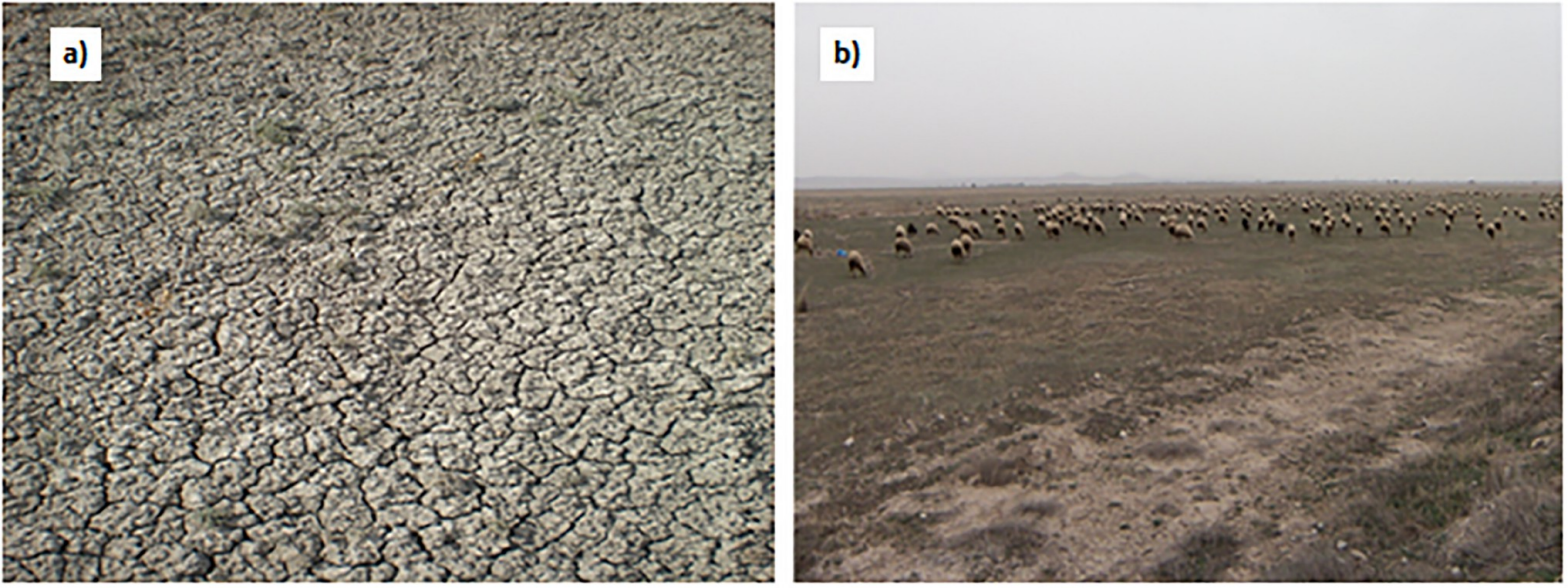
Sodium slickspot (a) and overgrazing in the study area (b). (Pictures taken by Mesut Budak).

Detection of surface salinity using satellite images is based on the principle that salt crust and other salt deposits without crusts on soil surface affect spectral reflection from soils [21]. In arid and semi-arid environments where evaporation is higher than precipitation, soluble salts in lower part of soil profile rise towards surface layer by capillarity and form thin white salt crusts. Salt crusts cause roughness on soil surface, causing differences in the reflection of electromagnetic energy [21]. Salt crusts, which are white in color, reflect a large part of the electromagnetic energy and create spectral signatures specific to saline areas; thus, saline surface soils can be determined with remote sensing [50]. The coefficient of variation (CV) for EC values in the study area is 90.5% [34]. High CV value of EC indicates that salinity is highly variable. Dispersed and dissolved humic substances in soil solution of sodic soils are deposited on soil surface and cause a dark surface color. The sodic soils are, therefore, termed as black sodic soils [49]. Dark surface color of saline-sodic soils in the study area decreased the reflactance from soil surface in contrast to the saline soils with salt crusts on surface (Fig 4a). Delavar et al. [21] suggested that sodium slickspot areas in smal scale depressions near Lake Urmia (Iran) cause roughness on soil surface and slickspots are considered an important source of error in determining salinity by remote sensing. In addition to masking impacts of humic substances, high variability of salinity and extansive sheep grazing in the study area prevented the formation of salt crusts on soil surface (Fig 4b).

Some of researchers indicated that spectral reflections may not provide adequate information in determining soil salinity of bare surface lands where salinity is <20 dS m^−1^ [1, 18]. Similarly, Mougenot et al. [51] stated that weak salt crystallization (salt content <10–15%) provides poor spectral descriptive properties in determining soil salinity using salinity indices. Therefoere, lack of common salt crusts and spectral confusion due to sodicity in the study area hampered the determination of soil salinity using salt indices with electromagnetic reflections in visible, NIR and SWIR wavelengths. In contrast to our study area, saline soils around Salt Lake in Turkey have been mapped using a highly correlated exponential regression model developed by using Landsat TM images and salinity indices [12]. The researchers indicated that high salt content of soils around the lake caused formation of widespread salt crusts, which reflect a large part of electromagnetic energy from surface. Therefore, salt indexes and spectral reflections had a high correlation, and a reliable regression model was developed to predict salinity of soils. The conclusion of Gorji et al. [12] supports our hypothesis that the relationship between spectral reflection and salt indexes is low due to insufficient salt crusts in our study area.

In addition to insufficient formation and heterogeneous distribution of salt crusts in the study area, other factors affecting electromagnetic energy reflected from soils are also responsible for low correlation. Bannari et al. [19] reported that organic matter content, moisture condition, texture, mineral content, roughness and vegetation cover of saline soils cause confusion in spectral signatures recorded by sensors. Verma et al. [52] stated high density of plant cover caused spectral confusion in determining soil salinity. The reseachers reported that vegetation hinders in obtaining spectral reflection information of soils. Similar to the salt indices, vegetation indices can also be used to monitor soil salinity. In contrast to the salt indices, vegetation indices provide more accurate results in areas where the surface is covered with dense vegetation [5, 53]. In contrast to the failure in assessing the salinity of the study area, Allbed et al. [16] successfully determined soil salinity of a highly saline land in the eastern part of Saudi Arabia using vegetation indices. The success in salinity assessment was attributed to the dense date palm trees on soil surface which improved spectral signature of vegetation. Halophytic plants in the study area had irregular distribution pattern on land surface. The field study was carried out at the driest part of the year; thus, the pasture was slightly covered with halophytic plants. The aforementioned causes might have contributed to the weakness in relationship between EC values measured and vegetation indices. Similar to our findings, previous studies [22, 54, 55] carried out to determine soil salinity using vegetation indices reported that these indices were unsuccesful to determine soil salinity in areas covered with species differing in salinity tolerance.

Spatial resolution of satellite images is an important factor in determining and mapping salinity [14, 20, 56]. Therefore, Ahmed and Andrianasolo [57] revealed that soil salinity can be mapped in more detailed using SPOT XS images compared to Landsat TM images. Therefore, moderate spatial resolution of TM images used in this study can be associated with the poor correlation between soil EC values and independent variables.

The highest correlation between soil EC and TM bands and image-derived from indices was obtained with SWIR1 wavelength (r = -0.437), followed by MSI (r = -0.423) and NDWI indices (r = 0.421) (Table 5). The level of this relationship was moderate in detailed distinguishing and mapping of soil salinity, however, the ANOVA test revealed that the models developed cannot provide a reliable salinity estimation for unsampled locations (Table 7). The MSI and NDVI indices were also correlated close to SWIR wavelength. Farifteh et al. [58] reported that moisture stress conditions can be used as an indirect indicator for determining salinity in saline alkaline soils. Dark color patches due to sodicity on the surface might have been perceived as moist surfaces, and thus, MSI and NDWI indexes may have a close relationship with SWIR wavelength. The TM image provides good results in determining the surface salinity by defining the absorption peaks in the SWIR band for flat and bare lands where evaporite minerals are dominant [59]. Contrary to these researchers, some other studies indicated that VIS and NIR wavelengths are more effective in determining soil salinity compared to SWIR wavelengths [23, 60].

Judkins and Myint [20] reported that determining and mapping variability of surface soil salinity using remote sensing are difficult. The researchers argued that determination of salinity is very complex, since the spectral range of mineral species that cause soil salinity does not have a single defining signature and that the surface cover causes a confusion in the spectral responses associated with surface salt deposits. In addition to the heterogeneity of surface conditions and the spatial variability of soil EC values, the sodicity also prevented success in determining the surface soil salinity using Landsat TM bands and the salt and vegetation indexes.

## Conclusion

The results revealed that salinity indices can be used in bare surface areas where salt is homogeneously distributed throughout the land and salt crust is common on surface with no sodicity problem. Slickspots were common in small-scale depressions of the study area. The slickspots caused roughness on soil surface and affected the reflectance from saline soil due to the dissolution of organic matter. In addition, vegetation indices may yield more successful results in saline areas with healthy vegetative cover on the surface. The results showed the importance of alternative techniques such as spectro-radiometry theory and methods, selection of appropriate spectral bands and considering the effect of environmental conditions to determine soil salinity using remote sensing. In addition, the results also indicated that Landsat 5 TM images with moderate spatial resolution do not provide sufficient spatial resolution to reveal soil salinity in patchy areas of large lands. High resolution images in similar fields would be more suitable to determine and map soil salinity. The spatial distribution of land degradation caused by salinity will be determined easily and policies and practices for the recreation of problematic areas can be implemented on time.

## Supporting information

S1 Dataset Minimal dataset of the study used for the interpretation of the results described in the manuscript. (XLS)
